# Supplementary material for: Association between oral microbiome and five types of respiratory infections: a two-sample Mendelian randomization study in east Asian population
Source: Front Microbiol. 2024 Apr 10;15:1392473. doi: 10.3389/fmicb.2024.1392473 (PMC11039966; doi:10.3389/fmicb.2024.1392473)
Supplement: Supplementary file 1 [file Data_Sheet_1.zip › Supplementary Materials/MR plots of saliva/Pneumonia/s__Actinomyces_oris_D_mgs_691/forest.pdf]

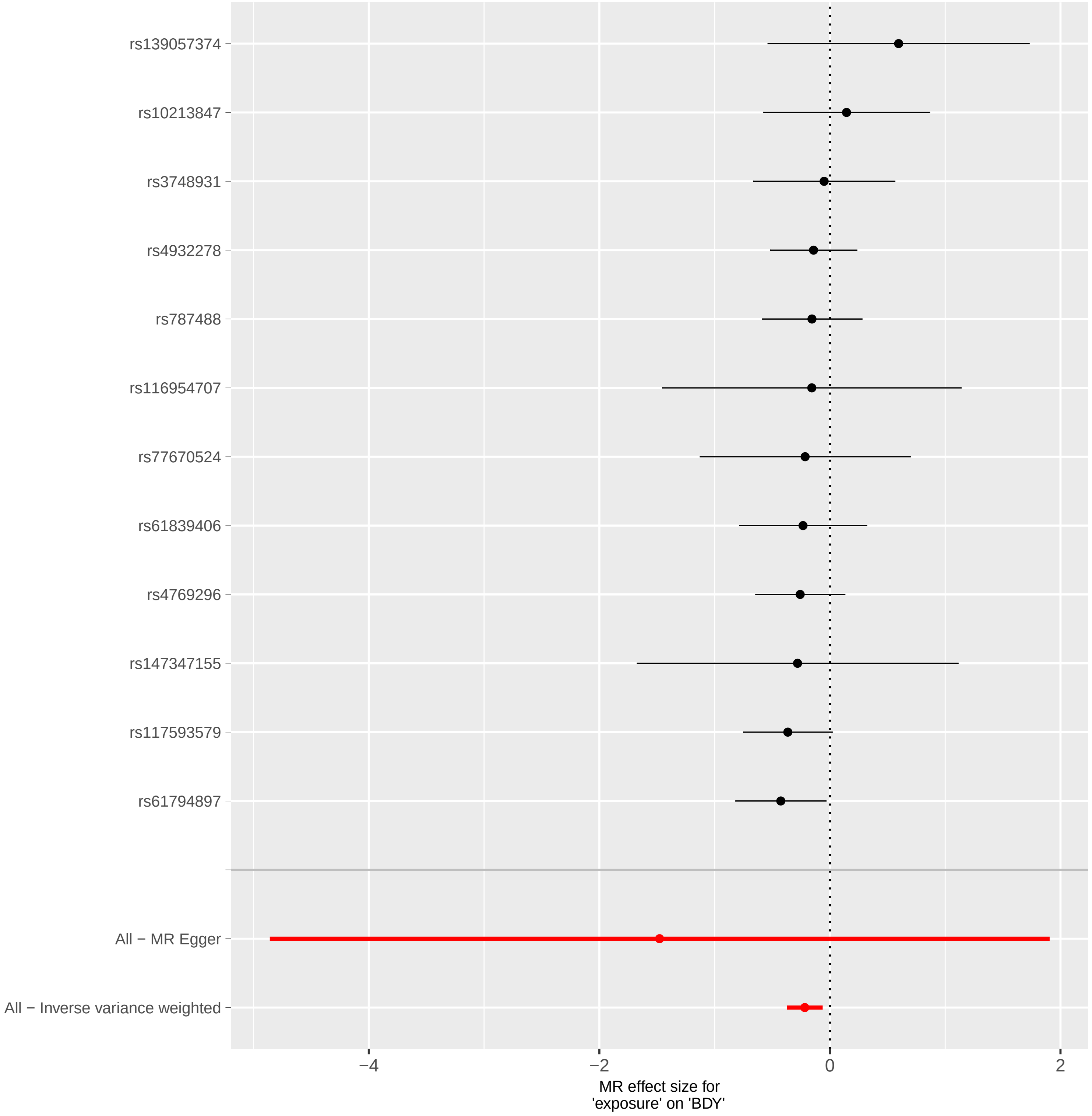
rs139057374
rs10213847
rs3748931
rs4932278
rs787488
rs116954707
rs77670524
rs61839406
rs4769296
rs147347155
rs117593579
rs61794897
All − MR Egger
All − Inverse variance weighted
−4
−2
0
2
MR effect size for
'exposure' on 'BDY'
